# Supplementary figures and images for: Functional analysis of lncRNAs based on competitive endogenous RNA in tongue squamous cell carcinoma
Source: PeerJ. 2019 May 28;7:e6991. doi: 10.7717/peerj.6991 (PMC6544013; doi:10.7717/peerj.6991)

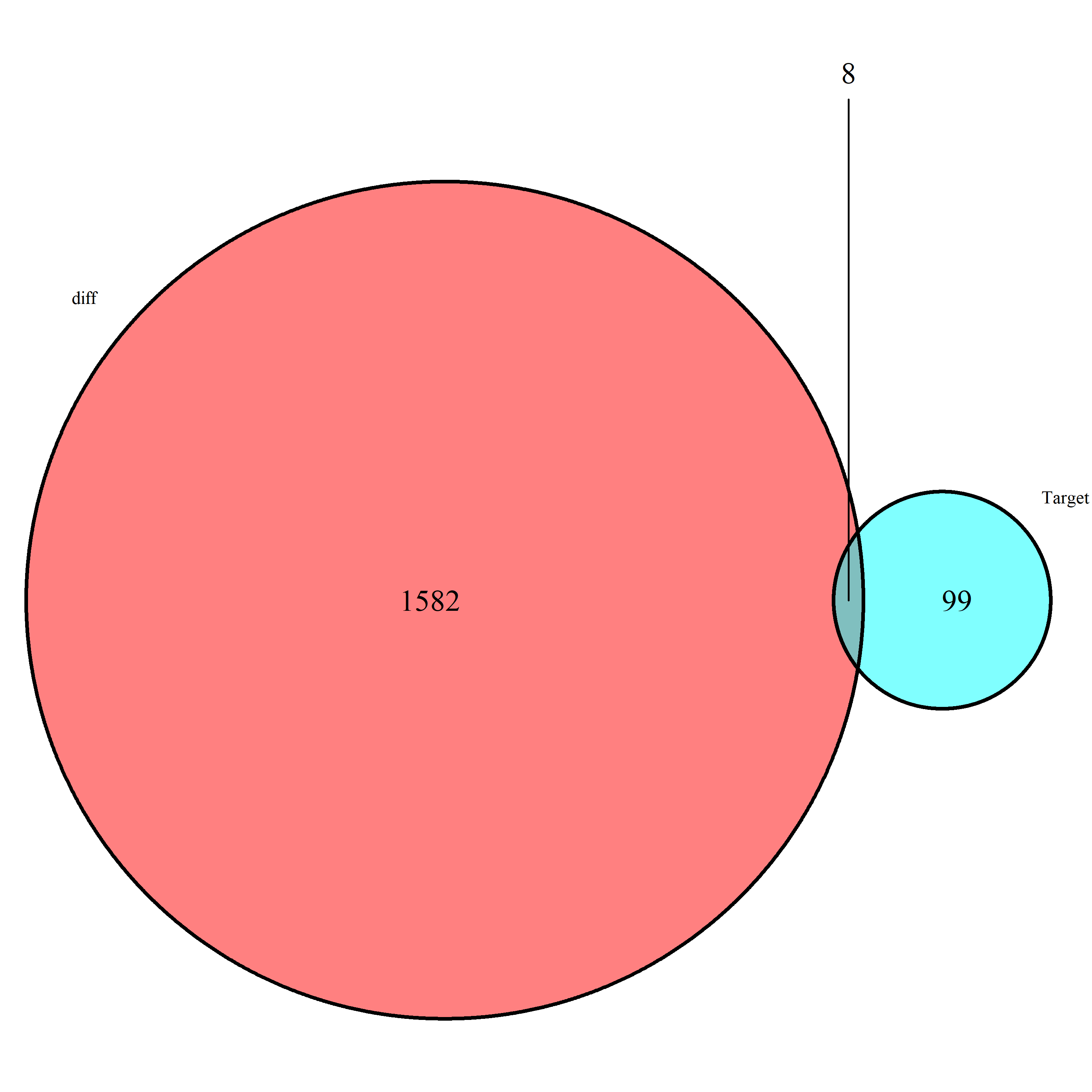

Supplement: Figure S1 — As shown in the figure, the number of mRNAs expressed in the red area is only the difference in expression. The blue area presents only the target number of mRNAs that are differentially expressed, while the purple area in the middle represents the number of mRNAs, which is both the differential expression and the target. [file peerj-07-6991-s007.tiff]
